# The Public Health Playbook: ideas for challenging the Corporate Playbook

**DOI:** 10.1093/eurpub/ckac129.092

**Published:** 2022-10-25

**Authors:** J Lacy-Nicols, R Marten, E Crosbie, R Moodie

**Affiliations:** 1 Melbourne School of Population and Global Health, University of Melbourne, Melbourne, Australia; 2 Alliance for Health Policy and Systems Research, WHO, Geneva, Switzerland; 3 School of Public Health, University of Nevada, Reno, USA

## Abstract

**Background:**

Many commercial actors use a range of coordinated and sophisticated strategies to protect business interests at the expense of public health, for example: attacking and undermining legitimate science, intimidating and vilifying critics, and framing and reframing discussion and debate. These strategies can be thought of as a ‘Corporate Playbook’ that spans numerous health and planet-harming industries. To counter this Corporate Playbook and advance health and wellbeing, public health actors need to develop, refine, and modernize their own Public Health Playbook.

**Methods:**

This paper seeks to consolidate thinking around how public health can counter and proactively minimize powerful commercial influences. It draws on previous attempts to develop approaches to counter commercial influence for public health, sustainability, human rights and democracy, and develops eight thematically grouped strategies.

**Results:**

We propose an initial eight strategies: 1) Expand the public health workforce and coalitions; 2) Increase public sector resources; 3) Link with and learn from social movements to foster collective solidarity; 4) Protect public health advocates from industry threats; 5) Develop and implement rigorous conflict of interest safeguards; 6) Monitor and expose corporate activities; 7) Debunk corporate arguments; and 8) Leverage diverse commercial interests.

**Conclusions:**

This set of strategies seeks to amplify inherent assets of the public health community and create opportunities to explicitly counter the Corporate Playbook. These strategies are not exhaustive, and our aim is to provoke further discussion and exploration on this topic. Moving forward, there is a need for further work to develop a rigorously researched and tested Public Health Playbook.

